# Reconstitution of Phase-Separated Signaling Clusters and Actin Polymerization on Supported Lipid Bilayers

**DOI:** 10.3389/fcell.2022.932483

**Published:** 2022-07-22

**Authors:** Xiaohang Cheng, Maria F. Ullo, Lindsay B. Case

**Affiliations:** Department of Biology, Massachusetts Institute of Technology, Cambridge, MA, United States

**Keywords:** phase separation, supported lipid bilayer, actin, ARP2/3 complex, total internal reflection fluorescence microscopy, biochemical reconstitution

## Abstract

Liquid–liquid phase separation driven by weak interactions between multivalent molecules contributes to the cellular organization by promoting the formation of biomolecular condensates. At membranes, phase separation can promote the assembly of transmembrane proteins with their cytoplasmic binding partners into micron-sized membrane-associated condensates. For example, phase separation promotes clustering of nephrin, a transmembrane adhesion molecule, resulting in increased Arp2/3 complex-dependent actin polymerization. *In vitro* reconstitution is a powerful approach to understand phase separation in biological systems. With a bottom-up approach, we can determine the molecules necessary and sufficient for phase separation, map the phase diagram by quantifying de-mixing over a range of molecular concentrations, assess the material properties of the condensed phase using fluorescence recovery after photobleaching (FRAP), and even determine how phase separation impacts downstream biochemical activity. Here, we describe a detailed protocol to reconstitute nephrin clusters on supported lipid bilayers with purified recombinant protein. We also describe how to measure Arp2/3 complex-dependent actin polymerization on bilayers using fluorescence microscopy. These different protocols can be performed independently or combined as needed. These general techniques can be applied to reconstitute and study phase-separated signaling clusters of many different receptors or to generally understand how actin polymerization is regulated at membranes.

## 1 Introduction

Liquid–liquid phase separation driven by weak interactions between multivalent molecules contributes to the cellular organization by promoting the formation of biomolecular condensates (Shin and Brangwynne, 2017). At membranes, phase separation can promote the assembly of transmembrane proteins with their cytoplasmic binding partners into micron-sized membrane-associated condensates (Case et al., 2019a). *In vitro* reconstitution is the experimental process of reverse engineering biological systems from purified components and is a useful approach to characterize biological phase separation. A bottom-up approach can enable scientists to determine the molecules necessary and sufficient for phase separation, map the phase diagram by quantifying de-mixing over a range of buffer conditions and molecular concentrations, assess the material properties of the condensed phase using fluorescence recovery after photobleaching (FRAP), and even determine how phase separation impacts downstream biochemical activity, such as actin polymerization (Su et al., 2016; Alberti et al., 2019; Case et al., 2019b; Huang et al., 2019).

Supported lipid bilayers can be used to reconstitute membrane-associated condensates from purified components. Typically, the cytoplasmic domain of a transmembrane protein is attached to a fluid lipid bilayer and cytoplasmic adaptor proteins are added to the solution. Interactions between the adaptor proteins and the receptor can promote phase separation to form micron-sized condensates on the membrane that can be visualized with total internal reflection fluorescence (TIRF) microscopy. *In vitro* reconstitution has been used to study the role of phase separation in many plasma membrane-associated condensates including nephrin clusters (Banjade and Rosen, 2014; Case et al., 2019b), LAT clusters (Su et al., 2016; Huang et al., 2019), integrin clusters (Case et al., 2022), post synaptic density condensates (Zeng et al., 2018), and growth factor receptor clusters (Lin et al., 2022). Reconstitution has also been used to study intracellular membrane-associated condensates (Banjade et al., 2022; Snead et al., 2022).

Traditional techniques to quantify actin polymerization *in vitro*, such as pyrene actin assays or ultracentrifugation, are limited to assessing the polymerization that occurs in solution (Cooper and Pollard, 1982; Doolittle et al., 2013a). However, in cells actin polymerization often occurs on membrane surfaces. For example, many nucleation-promoting factors of the Arp2/3 complex are associated with and are activated at membranes (Kim et al., 2000; Prehoda et al., 2000; Takenawa and Suetsugu, 2007). A microscopy-based approach to quantify actin polymerization on supported lipid bilayers enables scientists to dissect the specific mechanisms that may regulate actin nucleation and actin polymerization at membrane surfaces. In this study, we outline methods and procedures to make supported lipid bilayers, reconstitute membrane-associated condensates on bilayers, and quantify Arp2/3-dependent actin polymerization from membrane-associated condensates.

## 2 Materials and Equipment

Detailed materials needed for each method are outlined in Tables 1–4. These procedures will also require a chemical hood, an oven for drying glassware, a benchtop ultracentrifuge, and a total internal reflection fluorescence microscope with photobleaching capabilities. The purification of proteins will require access to shakers for bacterial growth, chromatography systems, and compatible columns.

**TABLE 1:** Reagents for cleaning glass vials. We have listed the specific items we have used to perform these assays. Many chemicals and supplies have numerous options available from multiple companies.

Reagent	Source	Identifier
Hellmanex™ III Liquid Cleaning Concentrate	Fisher Scientific	Cat# 14-385-864
NaOH	Millipore Sigma	Cat# 221465
MilliQ H_2_O	Multiple	NA
Glass vials for mixing lipids	Fisher Scientific	Cat# 03-391-7B
Glass vials for storing lipids	Avanti Polar Lipids	Cat# 6005111EA
Microwave	Multiple	NA

**TABLE 2:** Reagents for cleaning 96-well glass-bottom plates. Many chemicals and supplies have numerous options available from multiple companies. We have listed some of the specific items we have used to perform these assays.

Reagent	Source	Identifier
Hellmanex™ III Liquid Cleaning Concentrate	Fisher Scientific	Cat# 14-385-864
NaOH	Millipore Sigma	Cat# 221465
milliQ H_2_O	Multiple	NA
Glass bottom 96 well plates	Brooks Life Sciences System	Cat# MGB096-1-2-LG-L
1L Beaker	Multiple	NA
Stir Bar	Multiple	NA
Stirring hot plate	Multiple	NA
Microwave	Multiple	NA
Adhesive PCR plate seal	Fisher Scientific	Cat # AB-0626

**TABLE 3:** Reagents for preparing small unilamellar vesicles (SUVs). Many chemicals and supplies have numerous options available from multiple companies. We have listed some of the specific items we have used to perform these assays.

Reagent	Source	Identifier
16:0-18:1 PC (POPC) in chloroform	Avanti Polar Lipids	Cat# 850457C
18:1 DGS-NTA(Ni) in chloroform	Avanti Polar Lipids	Cat# 790404C
18:1 PEG5000 PE	Avanti Polar Lipids	Cat# 880230C
16:0-06:0 NBD PC	Avanti Polar Lipids	Cat# 810130C1MG
Chloroform	Millipore Sigma	Cat# 288306-100 ML
Hamilton Syringe (250 μ l volume)	VWR	Cat# 60376-668
Hamilton Syringe (10 μ l volume)	Fisher Scientific	Cat# 14-813-125
Vacuum Dessicator	Multiple	NA
Argon Gas (alternatively can use Nitrogen gas)	Airgas	Cat# AR HP200
PBS pH 7.3	Multiple	NA
Liquid nitrogen	Multiple	NA
Water bath sonicator (optional)	Multiple	NA

**TABLE 4:** Reagents for actin assembly assay. Many chemicals and supplies have numerous options available from multiple companies. We have listed some of the specific items we have used to perform these assays. We use actin purified from rabbit muscle (Doolittle et al., 2013a) and labeled with Alexa-647 C_2_ maleimide (Hansen et al., 2013). We use Arp2/3 purified from calf thymus (Doolittle et al., 2013b). The commercially available alternatives are listed here, but we have not directly tested our methods with these commercial reagents.

Reagent	Source	Identifier
Actin (>99% pure) from Rabbit Skeletal Muscle	Cytoskeleton, Inc.	Cat# AKL99
Actin protein (Rhodamine labeled)	Cytoskeleton, Inc.	Cat# AR05
Arp2/3 Protein Complex: Porcine Brain	Cytoskeleton, Inc.	Cat# RP01P
Capping protein (plasmid for protein expression in *e. coli*)	Addgene (plasmid)	Cat# 89950
ATP	Millipore Sigma	Cat# A2383
MgCl_2_	Millipore Sigma	Cat# M8266
EGTA	Millipore Sigma	Cat# E4378
KCl	Millipore Sigma	Cat# P9541
Hepes	Millipore Sigma	Cat# H3375
Glucose Oxidase	Millipore Sigma	Cat# G2133
Catalase from bovine liver	Millipore Sigma	Cat# C1345
Dextrose (D-Glucose)	Millipore Sigma	Cat# DX0145
BSA	Millipore Sigma	Cat# A3294
TCEP	Fisher Scientific	Cat# 58-056-11 ML

## 3 Methods

In this study, we provide step-by-step protocols to reconstitute phase-separated condensates and Arp2/3-dependent actin polymerization on supported lipid bilayers. We previously used these methods to determine how phase separation promotes the specific activity of N-WASP toward actin assembly (Case et al., 2019b). Several detailed methods studies have previously outlined similar approaches to study protein self-organization on supported lipid bilayers (Nguyen et al., 2015; Su et al., 2017; Ramm et al., 2018). The discussion section also provides additional information about alternative approaches and troubleshooting.

### 3.1 Cleaning Glass Vials

1. Prepare 400 ml of 3M NaOH in a glass bottle or glass graduated cylinder. Transfer to a clean 500 ml glass beaker.
2. Add glass vials to the NaOH solution. Add the vials one-by-one to ensure they fill with the solution and there are no trapped air bubbles. We typically wash ∼20 vials at a time.
3. Incubate 45 min at room temperature.
4. Fill two 500 ml beakers with 400 ml MilliQ H_2_O.
5. Rinse vials four times in MilliQ H_2_O by transferring back and forth between the two 500 ml beakers. Add the vials one-by-one to ensure they fill with the H_2_O and there are no trapped air bubbles. Repeat three times, replacing beakers with fresh water each time.
6. Fill a 500 ml beaker with 5% Helmanex in MilliQ H_2_O. Microwave 30–45 s to bring solution close to 40°C (i.e., warm to the touch). Add the vials one-by-one to ensure they fill with the solution and there are no trapped air bubbles. Incubate on a hot plate set to 50°C for 1 h. The solution should stay warm, but it does not need to be exactly 50°C.
7. Rinse vials six times in MilliQ H_2_O by transferring back and forth between the two 500 ml beakers filled with water. Add the vials one-by-one to ensure they fill with the H_2_O and there are no air bubbles. Repeat five times, replacing beakers with fresh water each time. You can rinse and reuse the beakers from step 5.
8. Empty the glass vials one-by-one and gently place them into a clean, dry, empty 500 ml beaker. Cover with foil and bake at 200°C for at least 3 h or overnight using an oven for drying glassware.
9. The vials should be completely dry and can be stored covered with foil to protect them from dust for 6–12 months.

### 3.2 Cleaning 96-Well Glass Bottom Plates

1. Prepare 1 L of 5% Helmanex solution in a 1 L glass beaker. Microwave for 60–90 s until the solution is close to 40°C (i.e., warm to the touch).
2. Add a stir bar to the beaker and move to a hot-plate stirrer set to 55°C.
3. Slowly submerge a 96-well plate in the solution with the outside of the glass bottom facing the bottom of the beaker at a slight angle. The opening of wells should be facing up so that air bubbles can escape. Ensure there are no visible air bubbles within the wells, which could lead to inconsistent cleaning.
4. Stir at the slowest setting. The 96-well plate should spin with the stir bar at the same pace. This will help reduce the heat gradient and promote more uniform cleaning. Be careful that the stir bar does not forcefully hit the plate, as this can crack the glass.
5. Incubate for 3.5–4 h. Incubating longer gives better results but incubating too long can weaken the glue between the plastic and glass, causing your wells to leak during the experiment.
6. Wash with copious amounts of MilliQ H_2_O over a sink. Ensure that each well fills completely with H_2_O, dump out the H_2_O, and repeat at least 25 times.
7. Place a kimwipe on the bench. Gently tap both sides of the plate on the kimwipe to remove excess water droplets.
8. Dry the wells completely, well-by-well with Argon gas. The airflow should be strong enough to remove water droplets and evaporate all liquid.
9. Seal with adhesive PCR plate seal. A cleaned and sealed plate can be used for 1–2 months. However, if you notice a decrease in the quality or fluidity of the bilayers, clean a new plate.

### 3.3 Preparing Small Unilamellar Vesicles

Small unilamellar vesicles (SUVs) can be made from any lipid composition. For assessing phase separation driven by multivalent protein interactions, we recommend starting with 98% POPC +2% NiNTA +0.1% PEG5000-PE. This is a simple lipid mixture that will give rise to fluid lipid bilayers. Lipid mixtures can undergo temperature-dependent phase transitions, and considering the transition temperature of the lipids is helpful when optimizing a new lipid mixture (Small, 1986).

1. Bring necessary materials to a chemical hood. You will need one glass vial per lipid mixture, one additional glass vial for chloroform waste, one 250 μl Hamilton syringe, one 10 μl Hamilton syringe, chloroform, and lipids.
2. Clean the glass syringes using chloroform. To do this, first insert the needle of the 250 μl Hamilton syringe into the chloroform bottle. Hold the bottle upside down, ensure the needle is submerged in the liquid, slowly draw out ∼250 μl chloroform and transfer it into the waste vial. Repeat this four times to transfer a total of ∼1 ml chloroform to the waste vial. The 250 μl syringe is now clean.
3. Insert the needle of the 10 μl Hamilton syringe into the 1 ml chloroform in the waste vial. Slowly pipette up and down four times to clean the syringe. Use this technique to clean syringes with needles too fine to insert directly into the chloroform bottle.
4. Calculate the volume of each lipid you will combine. Lipid composition in the final mixture should be calculated by percentage in moles. Based on the molecular weight and concentration indicated by the lipid product information, volumes of each lipid to add to the mix can be determined (Table 5).
5. Use the Hamilton syringes to transfer lipids into a clean, empty glass vial. We recommend starting with the largest volume and then adding the smaller volumes. For example, we typically start with a lipid mix containing total of 4 µmole lipids (Table 5), we might add 119 μl POPC using the 250 μl syringe, followed by 8.5 μl NiNTA using the 10 μl syringe, followed by 3.1 μl NBD-PC using the 10 μl syringe, followed by 2.3 μl PEG5000-PE using the 10 μl syringe.
6. After all lipids are added, gently swirl the vial.
7. Optional: Rapidly evaporate the chloroform from the lipid mixture by directing a gentle flow of Argon gas over the lipid mixture. For safety reasons, only attempt this step if it can be done in the chemical hood. This minimizes the exposure of lipids to oxygen to prevent lipid oxidation.
8. Alternatively, chloroform can be evaporated overnight in a vacuum desiccator.
9. Regardless of the evaporation method, place the glass vial containing the lipid mixture in a vacuum desiccator for several hours or overnight to ensure that lipids are fully dehydrated.
10. Wash the Hamilton syringes. First, insert the needle of the 10 μl Hamilton syringe into the 1 ml chloroform in the waste vial. Slowly pipette up and down four times to clean the syringe.
11. Next insert the needle of the 250 μl Hamilton syringe into the 1 ml chloroform in the waste vial. Slowly pipette up and down four times to clean the syringe.
12. Perform a final wash of the 250 μl Hamilton syringe. Insert the needle of the 250 μl Hamilton syringe into the chloroform bottle. Hold the bottle upside down and slowly draw out ∼250 μl chloroform and deposit it into the waste vial.
13. Disassemble the needles from syringes and let dry overnight. We often leave disassembled syringes on the lab bench covered with kimwipes to protect them from dust. After drying, re-insert the needle back into the syringe for storage in the original Styrofoam syringe box.
14. The following day, remove lipids from the desiccator. The lipids will appear as a thin, white film at the bottom of your tube.
15. Resuspend lipids in PBS pH 7.3. We resuspend 3 mg lipids (4 µmole) in 1.5 ml PBS for final lipid concentration of ∼2 mg/ml. The buffer should be above the transition temperature of your lipid mixture.
16. Cover glass vial with parafilm or clean screw top and vortex for ∼15–30 s until the solution becomes cloudy. This increased turbidity indicates multilamellar vesicles are forming.
17. Optional: Incubate for 30–60 min vortexing occasionally.
18. Using a 1,000 μl pipette, transfer the mixed and resuspended lipids into 1.5 ml Eppendorf tubes. To avoid bubbles do not transfer the entire volume. You can aliquot lipids into multiple tubes, depending on your experimental needs. We recommend starting with 500–750 μl lipids per tube.
19. Direct a gentle flow of Argon gas into the tube over the lipid mixture for 5–10 s. The goal is to remove as much oxygen as possible to prevent NiNTA hydrolysis.
20. To generate SUVs, freeze-thaw your lipid mixture. Fill a liquid nitrogen-safe container with liquid nitrogen and set a heat block or water bath to 40°C. Rapidly freeze lipids in liquid nitrogen, then thaw with heat. To ensure lipids are fully frozen, leave the tubes in liquid nitrogen for at least 30 s. Repeat this 25–30 times. As the multilamellar vesicles fracture into SUVs the solution will begin to clear. Alternatives to this freeze-thaw method are discussed below in the results.
21. Optional: At this point, frozen aliquots can be stored at −80°C for ∼2 months. In our experience, 98% POPC +2% NiNTA can be stored for up to 2 months. However, SUVs made from other lipids mixtures may not be amenable to long-term storage. When you are ready to use a lipid aliquot from −80°C storage, you should repeat the freeze-thaw 20–25 times.
22. After the freeze-thaw cycle is complete, pellet any remaining large or multilamellar vesicles with ultra-centrifugation. We use a tabletop ultracentrifuge (Beckman Optima TL Ultracentrifuge with rotor TLA100 and Thickwall Polyallomer Tubes (Beckman Coulter Cat# 343621) at 35,000 rpm (47,334 g) for 45 min at 4°C. The supernatant should be transparent and will contain the SUVs.
23. Avoiding the pellet, carefully transfer the supernatant to a new 1.5 ml Eppendorf tube.
24. Direct a gentle flow of Argon gas into the tube over the lipid mixture for 5–10 s. The goal is to remove as much oxygen as possible to prevent lipid oxidation.
25. The SUV mixture can be stored at 4°C for up to 2 weeks. Each time you open the tube, repeat step 24 to remove oxygen before storage. If you notice a decrease in the quality or fluidity of your bilayers, freeze-thaw, and pellet a new aliquot. In our experience, 98% POPC +2% NiNTA can be stored for up to 2 weeks at 4°C. However, SUVs made from other lipids mixtures may not be amenable to storage and might need to be prepared fresh daily. The long-term stability of SUVs is highly dependent on the lipid species present in the SUV. Both oxidation and hydrolytic degradation can impact lipid stability (Grit and Crommelin, 1993). Lipid oxidation is dependent on the degree of lipid saturation. Typically, more unsaturated lipids will oxidize quicker. Lipid hydrolysis is dependent on many factors including the solution buffer and the properties of the lipid molecules.

**TABLE 5:** Example calculations for converting lipid moles to volume.

Lipid name	MW, g/mole	Stock concentration, mg/mL	Final (%) in moles	Moles to add	Volume to add (µl)	Product catalog
16:0-18:1 PC	760	25	98	3.92 µ mole	119.17	850457
NBD-PC	771.8	1	0.1	0.004 µ mole	3.1	810130
PEG5000-PE	5,797.100	10	0.1	0.004 µ mole	2.32	880230
DGS-NTA(Ni)	1,057.003	10	2	0.08 µ mole	8.457	790404

### 3.4 Performing Phase Separation Experiments

**FIGURE 1:**
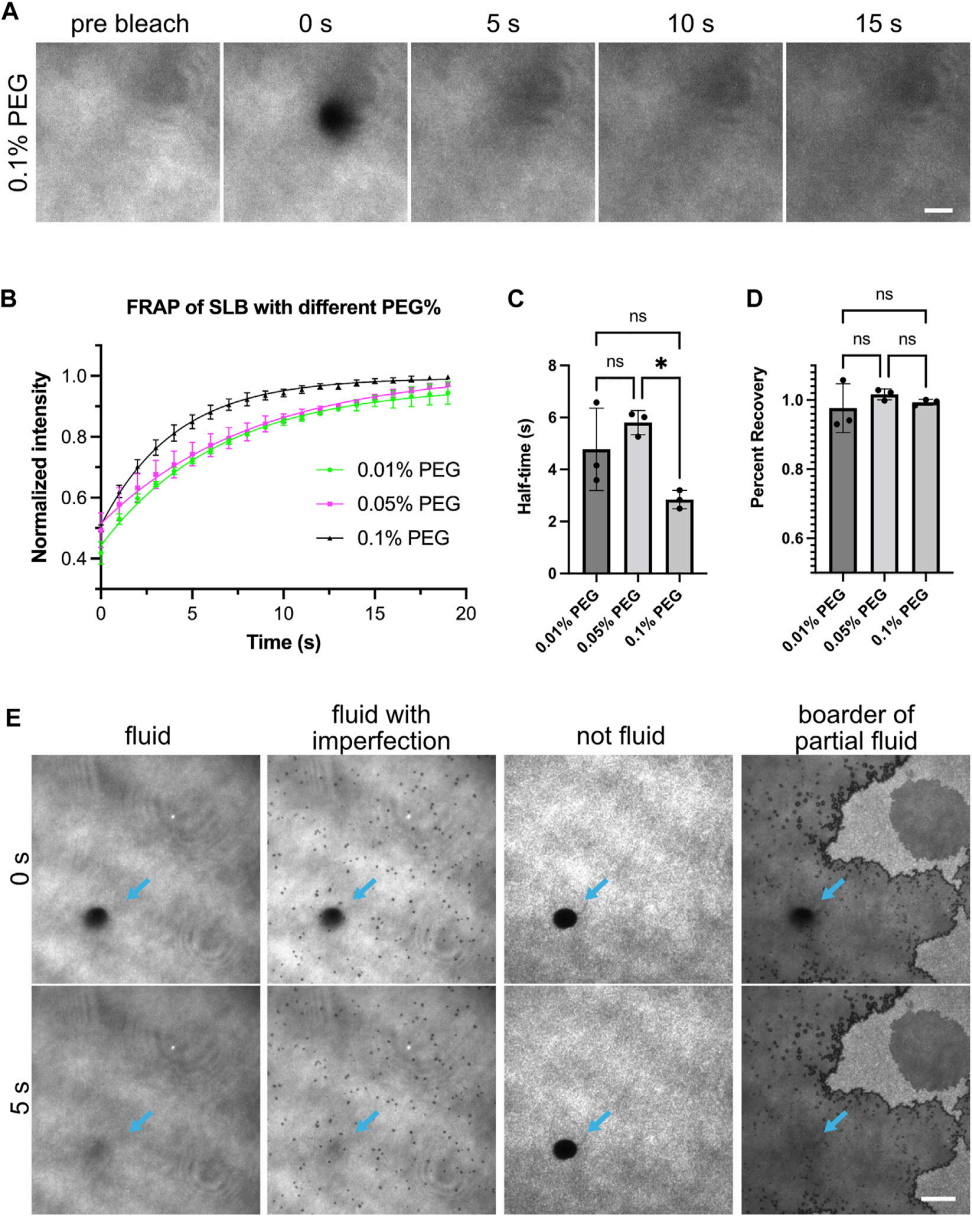
Fluidity of supported lipid bilayers. **(A)** Example images showing fluorescence recovery after photobleaching (FRAP) for lipid bilayers labeled by NBD-PC lipids. Bilayers imaged with Total Internal Reflection Fluorescence (TIRF) microscopy. This example bilayer contains 98% POPC, 2% Ni-NTA, 0.1% NBD-PC and 0.1% PEG5000-PE. Numbers above the images indicate seconds after photobleaching. A 5 µm-diameter region (outline can be seen in 0 s panel) is bleached with a 405 nm laser. Scale bar, 5 µm. **(B)** FRAP measurement of bilayer. Each point represents the mean ± standard deviation of three independent measurements. A curve calculated from a single exponential fit is overlaid in the graph. **(C,D)** Recovery half time and percentage recovery calculated from the single exponential fit for each measurement. Statistical analysis is carried out through ordinary one-way ANOVA with Tukey’s multiple comparisons test, * indicates the difference is significant at *p* < 0.05. **(E)** Examples of common bilayer defects. Blue arrows indicate the photobleached area. Scale bar, 10 µm.

1. On the day of the experiment, open the desired number of wells in the 96-well plate using a razor blade to carefully cut the foil away. You need one well for each replicate or experimental condition and an extra well for blank measurements. When first learning how to make supported lipid bilayers, we recommend starting with 4-6 wells. After getting some experience, you can increase the number of wells as necessary for the experiment. It is generally useful to prepare 1–2 extra wells in case of error.
2. Using a 1,000 μl pipette, add 300 μl MilliQ H_2_O to the well to wet the glass surface.
3. Remove the 300 μl of H_2_O.
4. Add 300 μl 6M NaOH to each well. NaOH will corrode the foil, so immediately clean any accidental drips with a kimwipe. 6M NaOH should be made fresh at least every 2 months to ensure maximum efficacy. We recommend making no more than 50 ml at a time in a 50 ml conical tube.
5. Cover the plate with the plastic lid and incubate on a hot plate set to 55°C for 1 h.
6. Carefully blot any evaporated liquid with a kimwipe. Then remove the 300 μl from the well with the pipette.
7. Add 300 μl 6M NaOH to each well and incubate for 1 h on a hot plate set to 55°C.
8. Carefully blot any evaporated liquid. Then remove the 300 μl from the well.
9. Wash wells three times with 500 μl MilliQ H_2_O. Pipette 500 μl H_2_O to each well, then immediately remove 500 μl from each well with the pipette. Repeat two times.
10. Wash wells three times with buffer. We often use 50 mM Hepes pH 7.25, 50 mM KCl, and 1 mM TCEP, which is also compatible with actin polymerization assays. You can optimize buffer conditions for your specific experimental needs. Both the proteins and the lipid bilayer may be sensitive to buffer properties such as pH, salt, and buffer species. To wash the wells pipette 500 μl buffer to each well, then immediately remove 500 μl from each well with the pipette. Repeat two times.
11. Remove any excess buffer from wells with a 200 μl pipette. Then add 200 μl buffer with a 200 μl pipette. From this point on, you must be very accurate and careful with your pipetting technique. We assume the volume in the well is 200 μl for all subsequent steps.
12. Add 15 µl SUVs to the well.
13. Incubate on a hotplate set to 50°C for 15 min. The SUVs will spontaneously fuse and collapse into a lipid bilayer when they touch the glass surface.
14. You now have a supported lipid bilayer (SLB) on the surface of the glass. You must take care to protect the bilayer and keep it intact for the duration of the experiment. To ensure the bilayer does not dehydrate, always keep at least 100 μl of buffer in the well. You must also be careful not to touch the bilayer with the pipette tip. When adding or removing liquid from the well, place the tip of the pipette toward the same corner of the well, so that any accidental contact is confined to one region of the bilayer. The pipette tip should contact the liquid without touching the bottom of the well. When pipetting, placing the neck of the pipette over the index finger of your non-dominant hand when adding or removing solutions into the well can reduce shaking and prevent accidental contact with the bilayer. Expel liquid slowly and gently to reduce agitation.
15. Rinse the bilayer three times to remove any remaining unfused SUVs. Remove 100 μl buffer from the well. Add 500 μl buffer to the well, gently pipette up and down once, then remove 500 μl buffer from the well. Repeat two times.
16. Add 100 μl buffer containing 1 mg/ml BSA. Incubate for 30 min at room temperature. Including 1 mg/ml BSA in the buffer prevents non-specific protein binding to surfaces. BSA is also a crowding agent that may influence phase separation. However, 1 mg/ml BSA is well below the concentrations typically used to induce molecular crowding, typically 100 mg/ml or higher.
17. If you are using multiple wells, you can leave the bilayers in this buffer for the rest of the day. We suggest staggering the addition of his-tagged protein to each well by at least 30 min. By staggering, you will ensure that subsequent incubation times are consistent between wells if you are doing an experiment that requires time.
18. Gather your protein and store it on ice. You will need purified recombinant protein to add to the bilayers. For this example, we used his8-nephrin, Nck, and N-WASP, and we have previously described their purification (Case et al., 2022; 2019b). Many different signaling clusters have been reconstituted using a similar approach (Su et al., 2016; Zeng et al., 2018; Huang et al., 2019; Lin et al., 2022). You may need to optimize the expression and purification of your specific proteins of interest.
19. Remove 100 μl from the well.
20. Add his-tagged protein to well. Prepare a solution of protein in buffer + 1 mg/ml BSA that is 2X the desired final concentration. Add 100 μl to all but one well and pipette up and down gently to mix the solution. The final well should only contain a bilayer and buffer (no protein or fluorophore) and will be used to measure background fluorescence on the microscope. You will need to optimize the concentrations necessary for desired density. We typically use 10 nM his8-tagged protein with 15% of proteins fluorescently labeled. With 2% NiNTA this gives ∼1,000–2000 molecules/micron^2^. We do not recommend using more than 15% fluorescently labeled protein to ensure fluorescence remains linear with density. See Section 3.5 for information on how to estimate protein density.
21. Incubate at room temperature for 1.5–2.0 h. Keep the plate covered with foil to avoid photobleaching.
22. Do not rinse away unbound protein until you are ready to perform the phase separation experiment. Rinse the bilayer three times to remove any unbound his-tagged protein. Remove 100 µl from the well. Add 500 µl buffer +1 mg/ml BSA to the well, gently pipette up and down once, then remove 500 µl buffer from the well. Repeat two times.
23. At this point, you should image the bilayers using TIRF microscopy. FRAP analysis of the his8-fluorescent protein should be used to ensure the bilayer is fluid (see Figure 1). Immobile bilayers should not be used for experiments (see troubleshooting).
24. Prepare the solution to induce phase separation. This could contain additional multivalent proteins, or perhaps a different salt concentration or pH. During protein purification, is important to remove any his-tags from proteins you are adding to the bulk solution, so they do not bind the Ni-NTA lipids. Also, the final purification step should be in a buffer containing betamercaptoethanol or TCEP, as DTT may reduce the nickel ions in the NiNTA lipids. Similarly, avoid the use of chelating agents such as EGTA or EDTA when using bilayers containing NiNTA lipids. Prepare a solution of protein in buffer + 1 mg/ml BSA +200 μg/ml glucose oxidase +35 μg/ml catalase +10 mM glucose + 1 mM TCEP that is 2X the desired final protein concentration. Add 100 μl to the well and gently pipette up and down 2 times to mix the solution. The glucose oxidase/catalase/glucose mixture will reduce photodamage from reactive oxygen species generated during fluorescence imaging.
25. Image clusters using TIRF microscopy. To visualize the formation of clusters, add proteins during time-lapse acquisition (see Figure 3). To assess clusters at steady-state, wait for 30 min-1 h before imaging. You should experimentally optimize this timing for your experimental system.
26. Image 15 random positions in the blank well with identical acquisition settings to your experiments. Use these images for background correction.

### 3.5 Estimating Protein Density on Bilayers

Single-molecule imaging with TIRF microscopy can be used to estimate the density of his-tagged proteins on supported lipid bilayers by combining picomolar concentrations of his-tagged protein labeled with one fluorophore (such as Alexa647) with nanomolar concentrations of the his-tagged protein labeled with a different fluorophore (such as Alexa488).

1. Prepare a solution of his-tagged protein (15% Alexa488, 0.0005% Alexa647, 85% unlabeled) in buffer + 1 mg/ml BSA that is 2X the desired final concentration. As above, add the his-tagged protein to the supported lipid bilayer at a range of concentrations. We recommend starting with concentrations between 5–500 nM.
2. Incubate at room temperature for 1.5–2.0 h. Keep the plate covered with foil to avoid photobleaching.
3. Rinse the bilayer three times to remove any unbound his-tagged protein. Remove 100 µl from the well. Add 500 µl buffer +1 mg/ml BSA to the well, gently pipette up and down once, then remove 500 µl buffer from the well. Repeat two times.
4. Image with TIRF microscopy in both channels. The Alexa488 channel can be used to assess the quality of the bilayer (i.e., is the bilayer uniform and fluid). The Alexa647 molecules should be sparse, which will enable the detection of single molecules. If the molecules are too dense or too sparse, you can optimize the concentration of Alexa647-labeled protein.
5. Acquire images of at least 15 random fields of view for each concentration.
6. The number of single molecules of Alexa647 can be calculated with ImageJ/FIJI (Schindelin et al., 2012; Schneider et al., 2012) using the TrackMate plugin with the DoG detector (Tinevez et al., 2017). You will need to optimize the algorithm parameters for your images.
7. Extrapolate the total density of his-tagged protein from the average density of Alexa647 molecules. For example, on a bilayer that contains 0.0005% Alexa647-labeled molecules, 50 molecules of Alexa647 is equivalent to 1 × 10^7^ total molecules.
8. Using this approach, you can determine the concentration of his-tagged protein that will give you the optimal membrane density. We recommend using the physiological membrane density if this has previously been determined experimentally in cells.
9. By plotting the calculated density versus the Alexa488 intensity, you can also determine an intensity-to-density conversion. If you keep imaging settings constant, this conversion can be used in future experiments to roughly estimate the density of molecules based on the his-tagged Alexa488 protein intensity.

### 3.6 Performing Arp2/3-Dependent Actin Assembly Assay

1. Prepare G-actin for your experiment. For long-term storage, we dialyze purified G-actin in G-buffer (2 mM Tris pH 8.0, 0.2 mM ATP, 0.1 mM CaCl_2_, 0.5 M DTT, 1 mM NaN_3_) at 4°C (Doolittle et al., 2013a). A few hours prior to an actin assembly assay, we change the dialysis buffer to a freshly made G-buffer. This will help reduce pre-formed seeds of F-actin.
2. Collect the reagents for actin assembly and store on ice (Table 4).
3. Prepare a stock solution of 10 mM MgCl_2_ + 10 mM EGTA in H_2_O.
4. Determine the volume of actin, labeled actin, Arp2/3, and capping protein you will need to add to the 200 μl reaction volume to reach desired final concentrations. We previously used 1 μ M actin (5% fluorescent-labeled), 3 nM Arp2/3 complex, and 6 nM capping protein (Case et al., 2019b). You may need to optimize concentrations based on the activity of your purified proteins (see Figure 5).
5. Take a two-color image of the experimental well as the “pre-actin” image. You should acquire an image of the clusters (e.g., using fluorescent N-WASP or Nephrin). You should also acquire an image in the actin channel. Since there is no actin added yet, this channel should be blank.
6. You will add the actin mixture directly to the 200 μl volume already in the well. Adding a large volume will dramatically change the protein concentrations, such as the concentration of Nck and N-WASP, which could impact their phase separation. You should confirm that this dilution will not affect phase separation prior to performing actin polymerization experiments. In our experience, changing the volume by less than 10% does not alter clustering or phase separation.
7. Combine actin and labeled actin in a new 1.5 ml Eppendorf tube. Add 2 μl of the 10 mM MgCl_2_/EGTA solution and pipette up and down gently to mix. This will replace Ca^2+^ with Mg^2+^ on G-actin monomers, which favors nucleation (Cooper et al., 1983). The concentration of MgCl_2_ and EGTA is currently over 1 mM, but when you add the polymerization mix to the experimental well (200 μl volume) the final concentration will be 0.1 mM MgCl_2_ and 0.1 mM EGTA. This lower EGTA concentration is important to prevent the chelation of the Ni-NTA lipids.
8. After 30 s, add 0.5 μl of 100 mM ATP, Arp2/3, and capping protein to the tube.
9. Quickly transfer to your experimental well. Pipette up and down to mix. Once you add Arp2/3 to the actin mixture, polymerization will begin. It is essential to move as quickly as possible.
10. Acquire a timelapse of actin polymerization. Ideally, you will take the first frame within a few seconds of adding the actin mixture to the well. You may need to optimize the imaging interval and duration of your experiment. We typically use 5 s intervals for 10 min. Actin polymerization is sensitive to photodamage, so it is critical to include glucose oxidase, catalase, and glucose in your reaction. If you notice less actin polymerization in the field of view compared with areas in the well that was not directly imaged, you should optimize the experiment to reduce photodamage.

## 4 Results

### 4.1 Characterization of Supported Lipid Bilayers

Fluid lipid bilayers are critical for observing liquid-liquid phase separation driven by interactions between receptors and adaptor proteins. If bilayers are immobile, proteins will not be able to diffuse and interact. Therefore, we first ensured the supported lipid bilayers were fluid by measuring the fluorescence recovery after photobleaching (FRAP) (Sprague and McNally, 2005). We incorporated 0.1% fluorescent NBD lipids to image the lipid bilayers directly (Figure 1). Fluid lipid bilayers recovered rapidly and exhibited near-full recovery after 15 s (Figure 1A). Including PEG-conjugated lipids can potentially increase membrane uniformity and membrane fluidity. We found that bilayers containing PEG5000-PE ranging from 0.01% to 0.1% concentration were all fluid, as exhibited by similar short FRAP half-time and near 100% recovery (Figures 1B–D). Common defects of supported lipid bilayers involve fluid bilayers with imperfections such as small holes, or non-fluid bilayers that show completely different morphology and FRAP profile (Figure 1E). Supported lipid bilayers with these defects should not be used for experiments. See the discussion for more tips on troubleshooting defective lipid bilayers.

**FIGURE 2:**
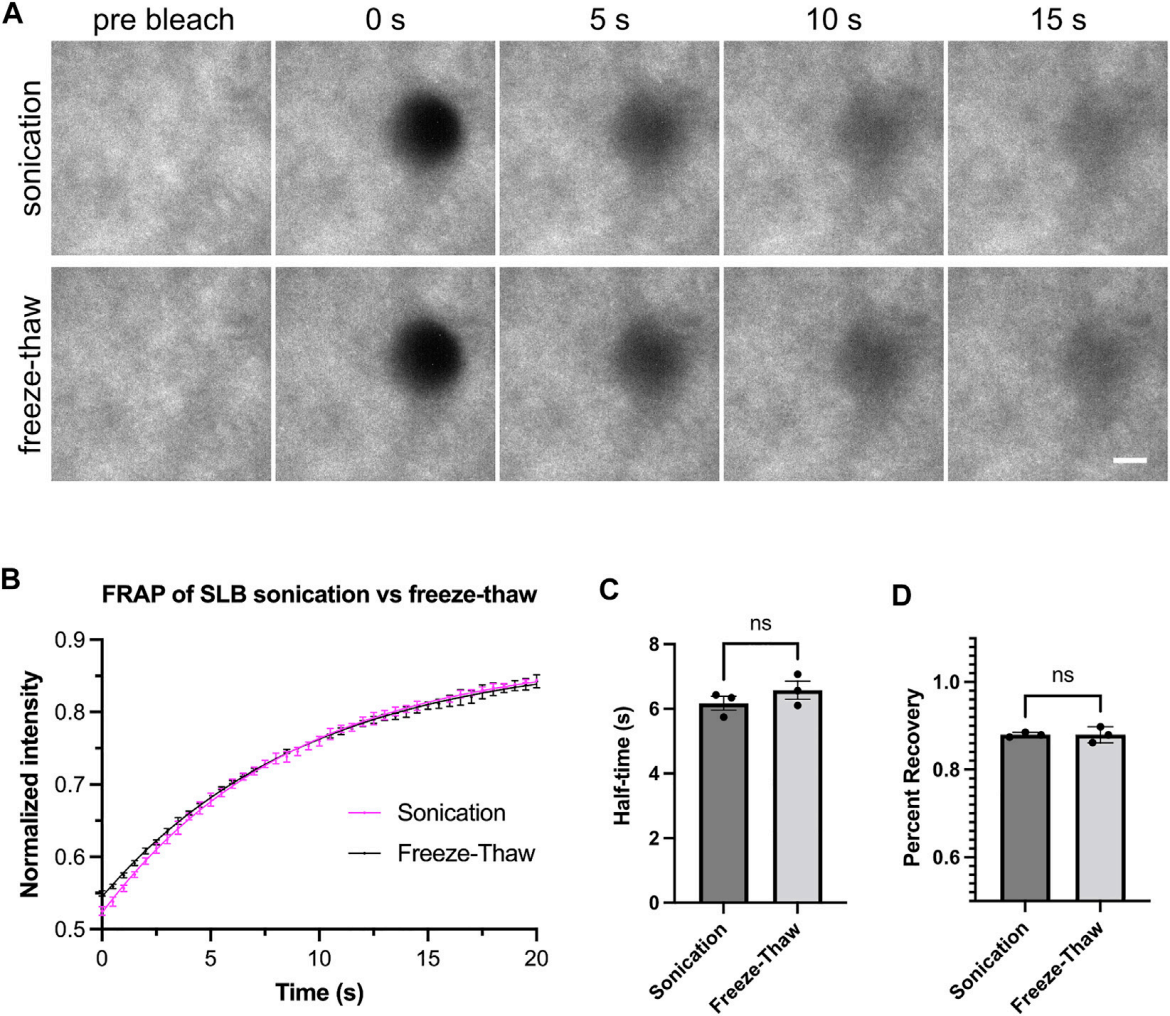
Freeze-thaw and sonication methods generate fluid lipid bilayers. **(A)** Example images showing fluorescence recovery after photobleaching (FRAP) for lipid bilayers labeled by His_8_-nephrin (15% Alexa405 labeled). Bilayers imaged with Total Internal Reflection Fluorescence (TIRF) microscopy. These bilayers contain 98% POPC, 2% Ni-NTA, and 0.01% PEG5000-PE. Scale bar, 5 µm. **(B)** FRAP measurement of bilayer. Each point represents the mean ± standard deviation of three independent measurements. A curve calculated from a single exponential fit is overlaid in the graph. **(C,D)** Recovery half time and percentage recovery calculated from the single exponential fit for each measurement. Statistical analysis is carried out through unpaired student *t*-test with significance at *p* < 0.05.

Our methods for generating Small Unilamellar Vesicles (SUVs) require many cycles of freezing and thawing (Section 3.3). This method is effective and does not require special equipment. However, freeze-thawing can be time-consuming, which could pose a problem when working with lipids that rapidly hydrolyze or are sensitive to oxidation (Grit and Crommelin, 1993). Therefore, we set out to test alternative methods to form SUVs. Low-frequency sonication and extrusion through a 100 nm pore-size filter have previously been used to generate SUVs (Ramm et al., 2018). We generated SUVs by low-frequency sonication in a water bath and performed ultracentrifugation after sonication to remove larger vesicles. After measuring the FRAP of bilayers from both SUV-generating methods, we found freeze-thaw and sonication resulted in comparably fluid membranes (Figure 2).

### 4.2 Characterization of Cluster Formation by Liquid–Liquid Phase Separation

**FIGURE 3:**
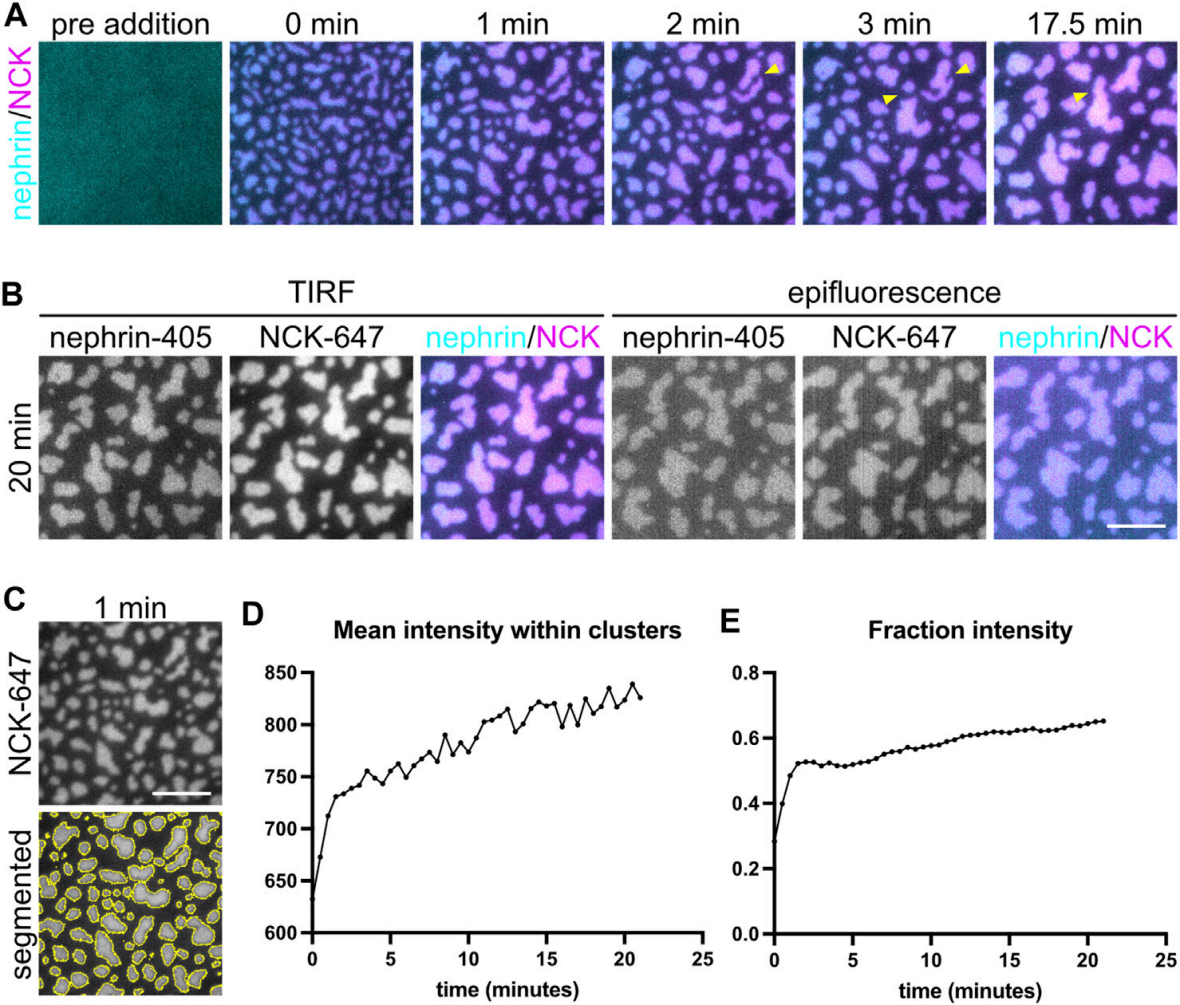
Cluster formation by liquid-liquid phase separation. **(A)** Time-lapse Total Internal Reflection Fluorescence (TIRF) images showing the formation of phase-separated clusters made by 2 µM Nck (15% labeled by Alexa647), 1 μM N-WASP with 10 nM His_8_-nephrin (15% labeled by Alexa405) on the membrane. Yellow arrowheads indicate example fusion events. **(B)** Comparison of TIRF and epifluorescence imaging for the clusters described in **(A)**. **(C)** Example time point at 1 min of cluster formation in NCK-647 channel (top) and the same image with the outline of the area above threshold for quantification (bottom). Scale bars, 5 µm. TIRF penetration depth is 110 nm for all channels. **(D–E)** Quantification of mean intensity within clusters **(D)** and fraction intensity of the clusters **(E)** over the period of time-lapse acquisition.

We next sought to induce clustering of biomolecular condensates through liquid–liquid phase separation using a previously published three-component system (Banjade and Rosen, 2014; Case et al., 2019b). Fluorescently labeled his8-tagged nephrin intracellular domain was attached to supported lipid bilayers containing 2% Ni-NTA lipids through nickel-histidine interactions. His8-tagged Nephrin was stably attached and uniformly distributed on the bilayer (Figure 3A, “pre addition”). Nck and N-WASP were added to the solution, and Nephrin immediately formed clusters upon their addition (Figure 3A, Supplementary Video S1). Individual clusters expanded and fused with other clusters over a 20-min period (Figure 3A yellow arrowheads, Supplementary Video S1). Since clustering occurs on membranes at the coverslip, it is ideal to image them using TIRF microscopy. While TIRF increases the signal-to-noise ratio enabling more accurate quantification, epifluorescence microscopy is sufficient to visualize clusters if TIRF is not available (Figure 3B).

### 4.3 Quantification of Cluster Images

When acquiring images of clusters, carefully select your imaging settings to ensure images are quantitative (Waters, 2009; Montero Llopis et al., 2021). You should optimize your imaging settings including the laser intensity, exposure time, and camera gain (if applicable), so that you use the appropriate dynamic range of your camera and do not saturate pixels. You should also acquire images of an empty well with identical settings to use for background subtraction. Finally, you should use the same imaging settings for all experimental conditions and replicates.

To characterize clusters, we quantified the intensity of molecules within clusters as well as the fractional intensity of the clustered region (Banjade and Rosen, 2014). To quantify the intensity of molecules within clusters, we first segmented the clusters. In ImageJ/FIJI this can be done by selecting, Image→Adjust →Threshold (Schindelin et al., 2012; Schneider et al., 2012). We adjusted the threshold parameters to accurately segment the clusters. ImageJ has several thresholding algorithms. We have had success with both the Triangle and Otsu algorithms, but you could try additional algorithms and optimize parameters for your images. Next, we measured the average intensity inside the segmented clusters (Figure 3C). In ImageJ, this can be done by selecting Analyze→Set Measurements and selecting “Mean gray value” and “Limit to threshold”. Then click Analyze→Measure to compute the mean intensity within the thresholded regions. Finally, we corrected these measurements by background subtraction. We calculated the Mean gray value of images of an empty well with no fluorophores. We then subtracted this background intensity from the measured cluster intensity (Figure 3D). If necessary, you can also correct images for uneven illumination using images of wells containing uniform fluorescent dyes. To quantify the fractional intensity of the clustered region, first we thresholded the clusters. Then we calculated the mean intensity of the thresholded image and subtracted the mean intensity of the background image. Using this corrected mean intensity, we calculated the corrected integrated intensity of the thresholded area by multiplying the area size and the corrected mean intensity. Next, we calculated the integrated intensity of the non-thresholded image and subtracted the integrated intensity of the background image. Finally, we divided the corrected integrated intensity of the thresholded image by that of the non-thresholded image (Figure 3E).

### 4.4 Reconstituting Actin Polymerization on Supported Lipid Bilayers

**FIGURE 4:**
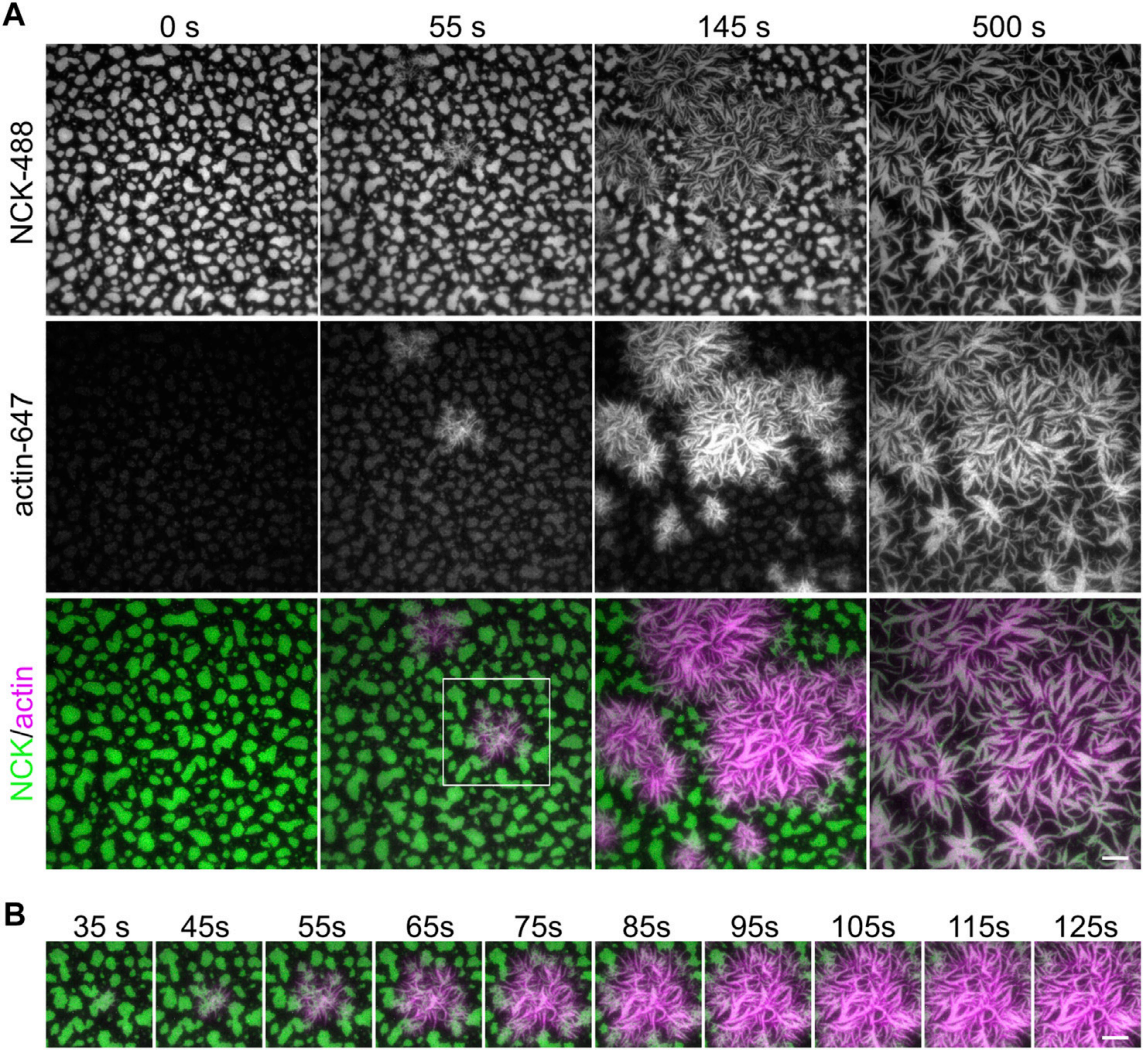
Actin polymerization from phase separated clusters. **(A)** Total Internal Reflection Fluorescence (TIRF) imaging of 1 µM actin (5% labeled by Alexa647, magenta) polymerization with 3 nM Arp2/3, 250 µM ATP, 0.1 mM MgCl_2_ and 0.1 mM EGTA, from clusters made from 500 nM Nck (15% labeled by Alexa488, green), 250 nM N-WASP and 2.5 nM His_8_-nephrin on the membrane. **(B)** Zoom-in view of region indicated by white box in **(A)**. Scale bars, 10 µm. Total Internal Reflection Fluorescence (TIRF) penetration depth 200 nm for all channels.

Actin polymerization can be initiated by the recruitment of Arp2/3-complex to Nephrin/Nck/N-WASP clusters (Case et al., 2019b). To initiate actin polymerization, we combined purified G-actin (5% Alexa647 fluorescently labeled), Arp2/3 complex, ATP, MgCl_2_, and EGTA with the pre-formed nephrin clusters (Section 3.5). TIRF imaging revealed actin polymerizing at nephrin/Nck clusters within seconds and continuing for minutes (Figures 4A,B, Supplementary Video S2). Moreover, Nck clusters began to take the shape of polymerized actin as the actin filaments continued to elongate (Figure 4A, Supplementary Video S2).

**FIGURE 5:**
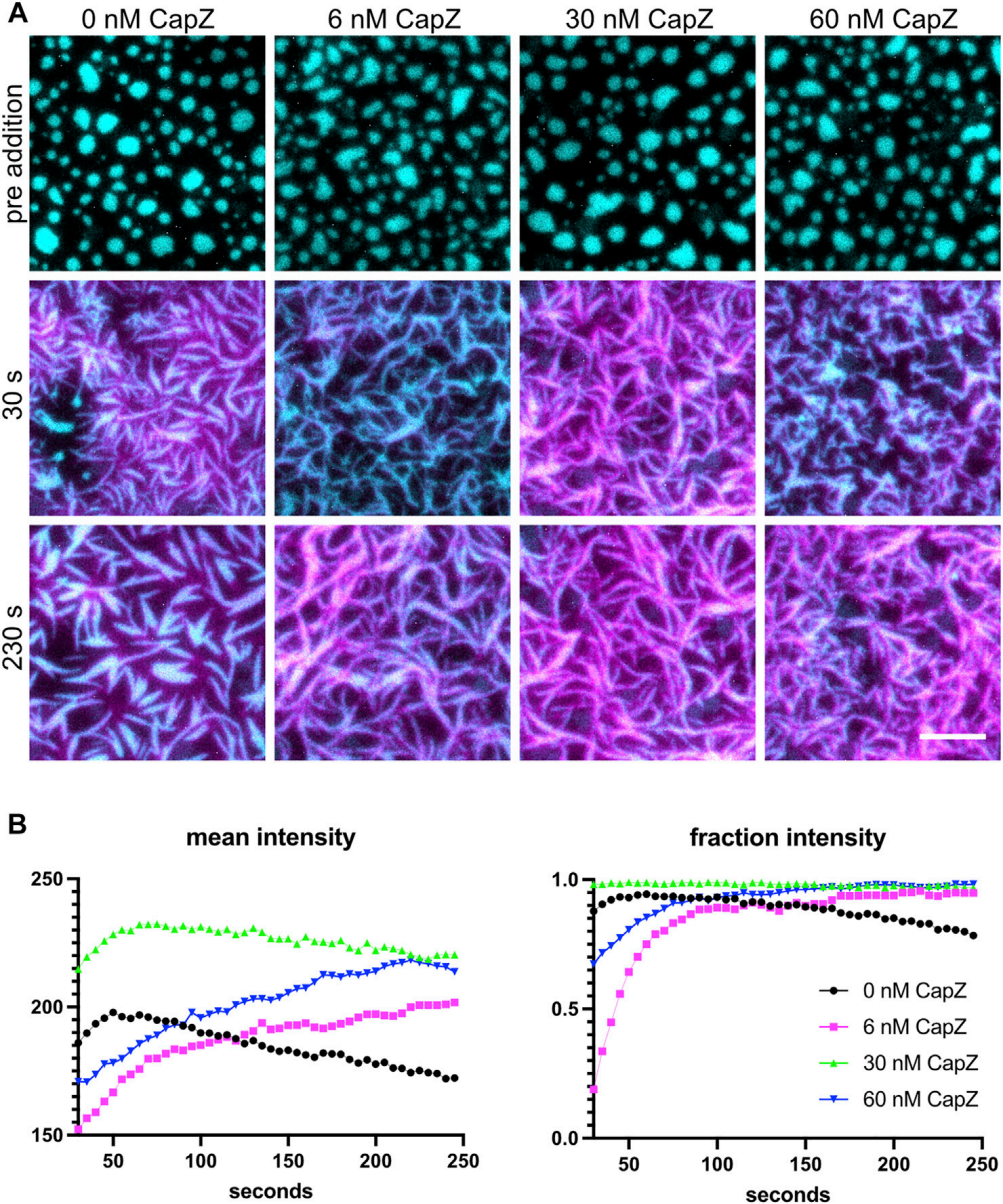
Actin polymerization with different levels of capping proteins. **(A)** 1 µM actin (5% labeled by Alexa647) polymerization is induced by 3 nM Arp2/3, 250 µM ATP, 0.1 mM MgCl_2_ and 0.1 mM EGTA, with different amounts of CapZ included in the system (indicated above each image), from clusters made from 500 nM Nck, 250 nM N-WASP and 1.25 nM His_8_-nephrin (15% labeled by Alexa405) on the membrane. Individual images are TIRF image overlays of nephrin-405 shown in cyan and actin-647 shown in magenta. Total Internal Reflection Fluorescence (TIRF) penetration depth 200 nm for all channels. Scale bar, 5 µm. **(B)** Quantification of actin mean and fraction intensity over time for the conditions showed in **(A)**. Actin was selected by selecting the intensity threshold between 250 and 65535 the individual 16-bit images, values for each frame are calculated as described in Section 4.4.

Polymerizing actin networks can be modulated by additional actin-binding proteins, such as capping protein. Actin polymerization occurs through both new filament nucleation and the elongation of existing filaments. Arp2/3 complex catalyzes the nucleation of a new daughter filament on the side of an existing mother filament while capping protein binds the ends of actin filaments and prevents elongation. Since capping protein both inhibits filament elongation and promotes Arp2/3-dependent nucleation (Akin and Mullins, 2008), including capping protein in the mix alters actin filament formation from the clusters (Case et al., 2019b). The actin polymerization rate and morphology of the F-actin network are altered when different concentrations of capping protein are used (Figure 5). Actin filaments grew out of the clusters and changed the shape of the clusters for all four capping protein concentrations tested. At lower concentrations of CapZ (6 nM), actin formed thin bundles that have a curly appearance, but actin bundles become shorter and thinner with higher CapZ concentrations (60 nM) (Figure 5A). We quantified the mean intensity of actin and fraction intensity of actin within clusters (Figure 5B) and found that changing CapZ concentration altered the dynamics of actin polymerization. Using different concentrations of Nephrin, Nck and N-WASP can change the actin polymerization rate and emergent cluster morphologies (Case et al., 2019b). Thus, the concentrations of Arp2/3 and capping protein should be optimized for specific experiments.

### 4.5 Quantification of Actin Polymerization Images

**FIGURE 6:**
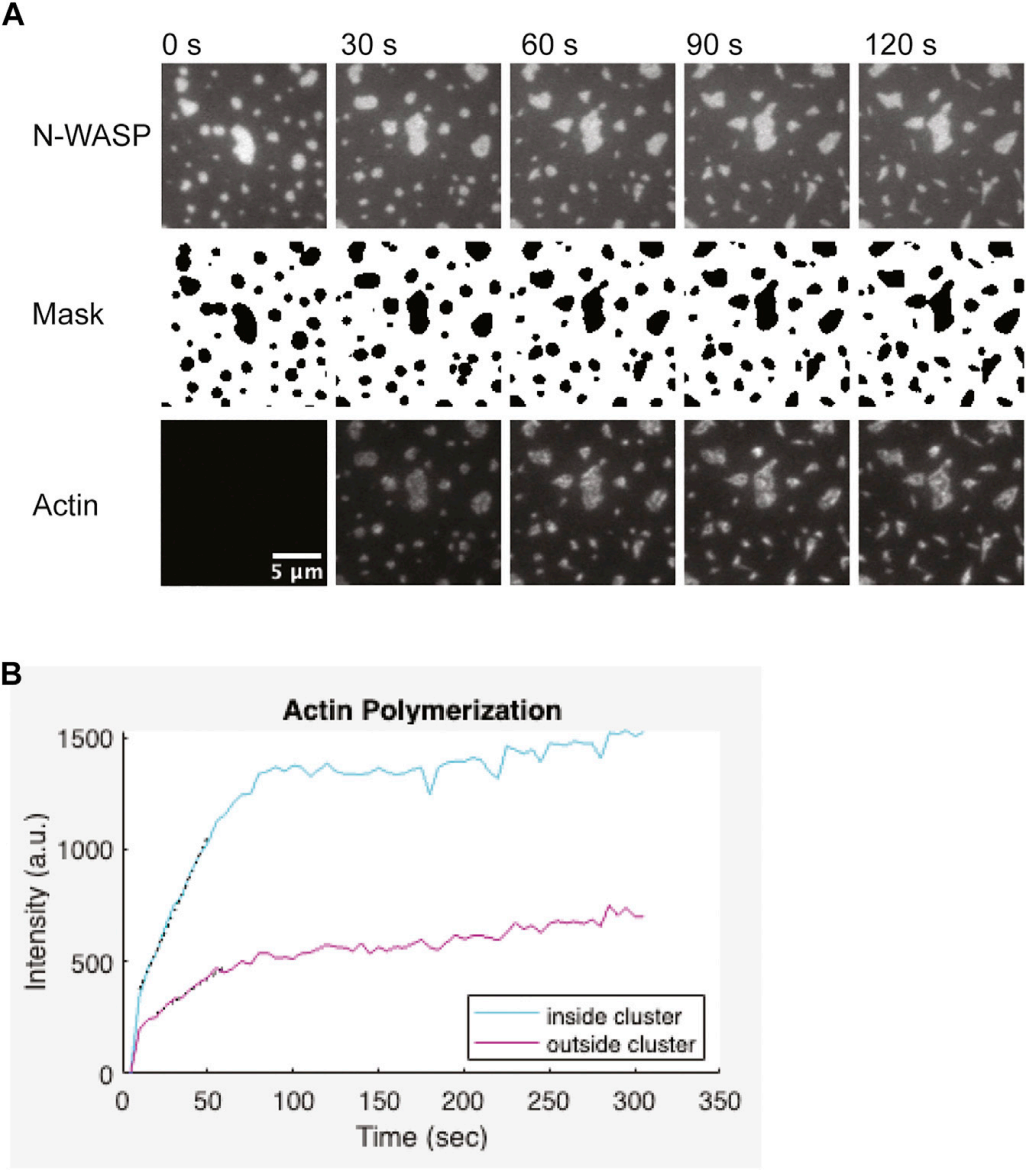
Representative actin polymerization analysis **(A)** 1 µM actin (5% labeled by Alexa647) polymerization is induced by 3 nM Arp2/3, 6 nM CapZ, 250 µM ATP, 0.1 mM MgCl_2_ and 0.1 mM EGTA, from clusters made from 1 µM Nck, 2 µM N-WASP (15% Alexa488) and 1.25 nM His_8_-nephrin on the membrane. Binary mask from segmentation of the N-WASP images. Pixels outside the clusters are white, pixels inside the clusters are black. Scale bar, 5 µm. **(B)** Quantification of actin polymerization over time for the condition showed in **(A)**. Graph was generated in Matlab. Black dotted lines indicate a linear fit of the five points around the half-max intensity.

The protocol outlined above (Section 3.6) describes how to capture a multi-channel timelapse image of Arp2/3-dependent actin polymerization from within membrane-associated clusters. One channel should be used to image fluorescent actin. A second channel should be used to image a molecule that localizes within clusters. This channel can be used to segment the image and determine the location of clusters (Figure 6A). This is important if you are interested in comparing the rate of actin assembly inside clustered regions to the rate in unclustered membrane regions. To quantify actin polymerization with TIRF microscopy, you must carefully select your imaging settings to ensure images are quantitative (Waters, 2009; Montero Llopis et al., 2021). You should optimize your imaging settings including the laser intensity, exposure time, and camera gain (if applicable) so that you use the appropriate dynamic range of your camera and do not saturate pixels. You should also acquire images of an empty well with identical settings to use for background subtraction. Finally, you should use the same imaging settings for all experimental conditions and replicates.

Depending on the concentrations of proteins used, actin filament elongation can deform the clusters. Quantification works best when clusters remain spatially separated from each other and do not dramatically change shape (Figure 6A). Thus, optimizing the correct balance of Arp2/3 complex and capping protein can help generate more easily quantifiable data. To measure the rate of actin assembly inside clusters, we first segmented the clusters by thresholding the images of the cluster molecule. The thresholded image was used to generate a binary mask of the clusters. This mask was applied to the actin channel to measure the intensity of actin inside clusters. To measure the intensity of actin outside clusters, we dilated and then inverted the mask before applying it to the actin channel. We repeated these measurements for each frame of the timelapse. Next, we used the same process to calculate the intensity inside and outside of clusters in the “pre-actin” image. We subtracted this background actin intensity from all actin intensity measurements. We plotted the background-corrected actin intensity versus time (Figure 6B). To estimate the rate of polymerization, we first determined the timepoint that the intensity reached half-maximum of the final actin intensity (t_1/2_). Then we performed a linear fit of the five datapoints between t_1/2_–2 timepoints and t_1/2_ + 2 timepoints. The quantification of actin polymerization can be done using ImageJ (Schindelin et al., 2012; Schneider et al., 2012), but we used custom Matlab code for our analysis. We provide the Matlab code and a compiled app to run the analysis as described (Cheng, 2022).

## 5 Discussion

The methods outlined here are meant to be as broadly applicable as possible. While we have outlined a multi-step protocol including making supported lipid bilayers, reconstituting phase separation, and measuring actin polymerization, you could also mix and match these methods to perform different experiments. For example, we have directly attached his8-tagged N-WASP to bilayers and measured actin polymerization in the absence of phase separation (Case et al., 2019b). It should also be possible to modify these methods to reconstitute formin-dependent actin polymerization on bilayers.

### 5.1 Troubleshooting

Working with supported lipid bilayers can be a technically challenging process. We have encountered many common problems, often due to the glass surface not being effectively cleaned. We summarize these problems and discuss potential solutions in Table 6.

**TABLE 6:** Troubleshooting guide.

Problem	Solution
Immobile bilayer (assessed by FRAP)	Glass surface is likely not ideally cleaned. Make a freshly cleaned plate and fresh 6M NaOH for the experiment. Could also try adding more PEG5000-PE to the lipid mixture
Holes in the bilayer	Not enough lipids or glass is not ideally cleaned. Make a freshly cleaned plate and fresh 6M NaOH for the experiment. Also, try increasing the number of SUVs you use to make the supported lipid bilayer
Aggregated his8-protein before clustering	Leaving his8-protein incubating on the bilayer for too long can lead to clustering (Section 3.4, Step 21). Also, adding too much his8-protein could induce Ni clustering. Try optimizing his8-protein concentration and incubation time. If your SUVs are older, this could lead to aggregation as well. Make fresh lipids
Testing new brand/model of well plates	Total surface area of the lipid bilayer per well may differ between different 96-well plates, thus influencing his8-protein density. Try titrating his8-protein to optimize concentration
Reduced phase-separated cluster formation or actin polymerization	Frequent fluorescent imaging may introduce photodamage and accumulation of reactive oxygen species. Leaving his8-protein incubating on the bilayer for too long can change the bilayer fluidity. Be consistent in your timing of all steps between different experiments and replicates. Try optimizing experiment sequences to avoid excessive excitation light

### 5.2 Alternative Glass Cleaning Methods

Several alternative procedures can be used when making supported lipid bilayers. For fluid-supported lipid bilayers, it is essential to use clean glass at every step of the preparation. Here we have outlined cleaning glass-bottom 96-well plates with 5% Helmanex followed by 6M NaOH. Cleaning glass coverslips with Piranha or RCA cleaning is more rigorous and effective (Nguyen et al., 2015). However, these solutions come with higher safety concerns and require working in a chemical hood. Furthermore, they are incompatible with the plastic-sided 96 well plates. The high number of wells afforded by these plates can be advantageous for certain experiments. A plasma cleaner can also be used to clean glass (Nguyen et al., 2015; Ramm et al., 2018). The method we describe using Helmanex and NaOH does not require additional equipment and is a nice compromise between ease, safety, and effectiveness. However, more rigorous glass cleaning methods could be considered if you are struggling to get consistent bilayers.

### 5.3 Alternative Methods to Attach Molecules to Bilayers

Here we describe using NiNTA lipids to attach his-tagged proteins to bilayers. The his-Nickel interaction attaches the protein close to the membrane. Since the his-tagged protein can dissociate from the membrane, the his-protein density on the membrane will decrease over time. An alternative method is to use biotinylated lipids to attach streptavidin-coupled molecules to the membrane. Biotin-streptavidin has a much higher binding affinity than histidine-nickel. Since streptavidin is also larger than a his8-tag, the attached molecule would be further from the membrane surface. Finally, maleimide lipids can be used to covalently attach a protein containing a single cysteine to a bilayer (Márquez et al., 2019).

### 5.4 Troubleshooting Actin Polymerization

In this protocol, we image fluorescent actin to assess actin polymerization. However, unlike in solution-based pyrene actin measurements (Doolittle et al., 2013a), both monomeric g-actin and polymerized f-actin will contribute to the measured actin intensity. Additional controls can be used to aid data interpretation and confirm that the measured rate of polymerization is due to Arp2/3-dependent nucleation. We recommend performing an experiment with 0 nM Arp2/3 complex to determine the contribution of fluorescent actin monomers and spontaneous Arp2/3-independent nucleation to the measured polymerization rate. If measurements are being done with TIRF microscopy, you should also confirm that polymerization is limited to the TIRF evanescent field by acquiring a z-stack of polymerized actin with confocal microscopy. Finally, ATP can inhibit the phase separation of some molecules (Patel et al., 2017), and phase separation is also sensitive to other changes in the buffer. We recommend performing a control experiment by adding MgCl_2_, EGTA, and ATP to clusters to confirm the polymerization buffer does not alter the degree of clustering. In our experiments, the final buffer concentrations of 0.1 mM MgCl_2_, 0.1 mM EGTA, and 250 μΜ ATP did not impact clusters.

## Data Availability

The raw data supporting the conclusions of this article are available as a dataset on datadryad.org. https://doi.org/10.5061/dryad.p8cz8w9ss.

## Supplementary Material

Supplementary Video S1 Phase separated cluster formation. Individual frames are TIRF microscopy images as shown in Figure 3. Image stack is processed with in Image Stabilizer plugin (Li, 2008) in FIJI for better illustration.

Supplementary Video S2 Actin polymerization from phase separated protein clusters. Individual frames are TIRF microscopy images as shown in Figure 4.

## References

[B1] Akin O. Mullins R. D. (2008). Capping Protein Increases the Rate of Actin-Based Motility by Promoting Filament Nucleation by the Arp2/3 Complex. Cell 133, 841–851. 10.1016/j.cell.2008.04.011 18510928 PMC2576297

[B2] Alberti S. Gladfelter A. Mittag T. (2019). Considerations and Challenges in Studying Liquid-Liquid Phase Separation and Biomolecular Condensates. Cell 176, 419–434. 10.1016/j.cell.2018.12.035 30682370 PMC6445271

[B3] Banjade S. Rosen M. K. (2014). Phase Transitions of Multivalent Proteins Can Promote Clustering of Membrane Receptors. eLife 3, e04123. 10.7554/eLife.04123 25321392 PMC4238058

[B4] Banjade S. Zhu L. Jorgensen J. R. Suzuki S. W. Emr S. D. (2022). Recruitment and Organization of ESCRT-0 and Ubiquitinated Cargo via Condensation. Sci. Adv. 8, eabm5149. 10.1126/sciadv.abm5149 35363519 PMC10938570

[B5] Case L. B. De Pasquale M. Henry L. Rosen M. K. (2022). Synergistic Phase Separation of Two Pathways Promotes Integrin Clustering and Nascent Adhesion Formation. eLife 11, e72588. 10.7554/eLife.72588 35049497 PMC8791637

[B6] Case L. B. Ditlev J. A. Rosen M. K. (2019a). Regulation of Transmembrane Signaling by Phase Separation. Annu. Rev. Biophys. 48, 465–494. 10.1146/annurev-biophys-052118-115534 30951647 PMC6771929

[B7] Case L. B. Zhang X. Ditlev J. A. Rosen M. K. (2019b). Stoichiometry Controls Activity of Phase-Separated Clusters of Actin Signaling Proteins. Science 363, 1093–1097. 10.1126/science.aau6313 30846599 PMC6784323

[B8] Cheng Xiaohang (2022). Reconstitution of Phase-Separated Signaling Clusters and Actin Polymerization on Supported Lipid Bilayers. Dryad Dataset. 10.5061/dryad.p8cz8w9ss PMC9361016 35959492

[B9] Cooper J. A. Buhle E. L. Walker S. B. Tsong T. Y. Pollard T. D. (1983). Kinetic Evidence for a Monomer Activation Step in Actin Polymerization. Biochemistry 22, 2193–2202. 10.1021/bi00278a021 6860660

[B10] Cooper J. A. Pollard T. D. (1982). “[19] Methods to Measure Actin Polymerization,” in Methods in Enzymology, Structural and Contractile Proteins Part B: The Contractile Apparatus and the Cytoskeleton (Cambridge, Unites States: Academic Press), 182–210. 10.1016/0076-6879(82)85021-0 6889668

[B11] Doolittle L. K. Rosen M. K. Padrick S. B. (2013a). Measurement and Analysis of *In Vitro* Actin Polymerization. Methods Mol. Biol. Clifton N. J. 1046, 273–293. 10.1007/978-1-62703-538-5_16 PMC3995334 23868594

[B12] Doolittle L. K. Rosen M. K. Padrick S. B. (2013b). “Purification of Native Arp2/3 Complex from Bovine Thymus,” in Adhesion Protein Protocols, Methods in Molecular Biology. Editor Coutts A. S. (Totowa, NJ: Humana Press), 231–250. 10.1007/978-1-62703-538-5_14 PMC3994882 23868592

[B13] Grit M. Crommelin D. J. (1993). Chemical Stability of Liposomes: Implications for Their Physical Stability. Chem. Phys. Lipids 64, 3–18. 10.1016/0009-3084(93)90053-6 8242840

[B14] Hansen S. D. Zuchero J. B. Mullins R. D. (2013). “Cytoplasmic Actin: Purification and Single Molecule Assembly Assays,” in Adhesion Protein Protocols, Methods in Molecular Biology. Editor Coutts A. S. (Totowa, NJ: Humana Press), 145–170. 10.1007/978-1-62703-538-5_9 PMC4013826 23868587

[B15] Huang W. Y. C. Alvarez S. Kondo Y. Lee Y. K. Chung J. K. Lam H. Y. M. (2019). A Molecular Assembly Phase Transition and Kinetic Proofreading Modulate Ras Activation by SOS. Science 363, 1098–1103. 10.1126/science.aau5721 30846600 PMC6563836

[B16] Kim A. S. Kakalis L. T. Abdul-Manan N. Liu G. A. Rosen M. K. (2000). Autoinhibition and Activation Mechanisms of the Wiskott-Aldrich Syndrome Protein. Nature 404, 151–158. 10.1038/35004513 10724160

[B17] Li K. (2008). The Image Stabilizer Plugin for ImageJ. Available at: http://www.cs.cmu.edu/~kangli/code/Image_Stabilizer.html (Accessed Februar, 2008).

[B18] Lin C.-C. Suen K. M. Jeffrey P.-A. Wieteska L. Lidster J. A. Bao P. (2022). Receptor Tyrosine Kinases Regulate Signal Transduction through a Liquid-Liquid Phase Separated State. Mol. Cell 82, 1089–1106.e12. 10.1016/j.molcel.2022.02.005 35231400 PMC8937303

[B19] Márquez I. Díaz-Haro G. Vélez M. (2019). Surface Orientation and Binding Strength Modulate Shape of FtsZ on Lipid Surfaces. Int. J. Mol. Sci. 20, 2545. 10.3390/ijms20102545 31137602 PMC6566678

[B20] Montero Llopis P. Senft R. A. Ross-Elliott T. J. Stephansky R. Keeley D. P. Koshar P. (2021). Best Practices and Tools for Reporting Reproducible Fluorescence Microscopy Methods. Nat. Methods 18, 1463–1476. 10.1038/s41592-021-01156-w 34099930

[B21] Nguyen P. A. Field C. M. Groen A. C. Mitchison T. J. Loose M. (2015). Using Supported Bilayers to Study the Spatiotemporal Organization of Membrane-Bound Proteins. Methods Cell Biol. 128, 223–241. 10.1016/bs.mcb.2015.01.007 25997350 PMC4578691

[B22] Patel A. Malinovska L. Saha S. Wang J. Alberti S. Krishnan Y. (2017). ATP as a Biological Hydrotrope. Science 356, 753–756. 10.1126/science.aaf6846 28522535

[B23] Prehoda K. E. Scott J. A. Mullins R. D. Lim W. A. (2000). Integration of Multiple Signals through Cooperative Regulation of the N-WASP-Arp2/3 Complex. Science 290, 801–806. 10.1126/science.290.5492.801 11052943

[B24] Ramm B. Glock P. Schwille P. (2018). *In Vitro* Reconstitution of Self-Organizing Protein Patterns on Supported Lipid Bilayers. J. Vis. Exp. JoVE. 137, 58139. 10.3791/58139 PMC6126581 30102292

[B25] Schindelin J. Arganda-Carreras I. Frise E. Kaynig V. Longair M. Pietzsch T. (2012). Fiji: an Open-Source Platform for Biological-Image Analysis. Nat. Methods 9, 676–682. 10.1038/nmeth.2019 22743772 PMC3855844

[B26] Schneider C. A. Rasband W. S. Eliceiri K. W. (2012). NIH Image to ImageJ: 25 Years of Image Analysis. Nat. Methods 9, 671–675. 10.1038/nmeth.2089 22930834 PMC5554542

[B27] Shin Y. Brangwynne C. P. (2017). Liquid Phase Condensation in Cell Physiology and Disease. Science 357, eaaf4382. 10.1126/science.aaf4382 28935776

[B28] Small D. M. (1986). The Physical Chemistry of Lipids: From Alkanes to Phospholipids, Handbook of Lipid Research, 4. New York: Plenum Press.

[B29] Snead W. T. Jalihal A. P. Gerbich T. M. Seim I. Hu Z. Gladfelter A. S. (2022). Membrane Surfaces Regulate Assembly of Ribonucleoprotein Condensates. Nat. Cell Biol. 24, 461–470. 10.1038/s41556-022-00882-3 35411085 PMC9035128

[B30] Sprague B. L. McNally J. G. (2005). FRAP Analysis of Binding: Proper and Fitting. Trends Cell Biol. 15, 84–91. 10.1016/j.tcb.2004.12.001 15695095

[B31] Su X. Ditlev J. A. Hui E. Xing W. Banjade S. Okrut J. (2016). Phase Separation of Signaling Molecules Promotes T Cell Receptor Signal Transduction. Science 352, 595–599. 10.1126/science.aad9964 27056844 PMC4892427

[B32] Su X. Ditlev J. A. Rosen M. K. Vale R. D. (2017). Reconstitution of TCR Signaling Using Supported Lipid Bilayers. Methods Mol. Biol. Clifton N. J. 1584, 65–76. 10.1007/978-1-4939-6881-7_5 PMC5633369 28255696

[B33] Takenawa T. Suetsugu S. (2007). The WASP–WAVE Protein Network: Connecting the Membrane to the Cytoskeleton. Nat. Rev. Mol. Cell Biol. 8, 37–48. 10.1038/nrm2069 17183359

[B34] Tinevez J.-Y. Perry N. Schindelin J. Hoopes G. M. Reynolds G. D. Laplantine E. (2017). TrackMate: An Open and Extensible Platform for Single-Particle Tracking. Methods, Image Process. Biol. 115, 80–90. 10.1016/j.ymeth.2016.09.016 27713081

[B35] Waters J. C. (2009). Accuracy and Precision in Quantitative Fluorescence Microscopy. J. Cell Biol. 185, 1135–1148. 10.1083/jcb.200903097 19564400 PMC2712964

[B36] Zeng M. Chen X. Guan D. Xu J. Wu H. Tong P. (2018). Reconstituted Postsynaptic Density as a Molecular Platform for Understanding Synapse Formation and Plasticity. Cell 174, 1172–1187.e16. 10.1016/j.cell.2018.06.047 30078712

