# Corrigendum: Reconstitution of phase-separated signaling clusters and actin polymerization on supported lipid bilayers

**DOI:** 10.3389/fcell.2023.1274775

**Published:** 2023-08-18

**Authors:** Xiaohang Cheng, Maria F. Ullo, Lindsay B. Case

**Affiliations:** Department of Biology, Massachusetts Institute of Technology, Cambridge, MA, United States

**Keywords:** phase separation, supported lipid bilayer, actin, ARP2/3 complex, total internal reflection fluorescence microscopy, biochemical reconstitution

In the published article, there was an error in the captions for **Figures 4–6** as published. In these captions, “0.25 µM” should be “250 µM.” The corrected captions appears below.

“FIGURE 4 | Actin polymerization from phase separated clusters. **(A)** Total Internal Reflection Fluorescence (TIRF) imaging of 1 µM actin (5% labeled by Alexa 647, magenta) polymerization with 3 nM Arp2/3, 250 µM ATP, 0.1 mM MgCl_2_ and 0.1 mM EGTA, from clusters made from 500 nM Nck (15% labeled by Alexa488, green), 250 nM N-WASP and 2.5 nM His_8_-nephrin on the membrane. **(B)** Zoom-in view of region indicated by white box in **(A)**. Scale bars, 10 µm. Total Internal Reflection Fluorescence (TIRF) penetration depth 200 nm for all channels.”

“FIGURE 5 | Actin polymerization with different levels of capping proteins. **(A)** 1 µM actin (5% labeled by Alexa647) polymerization is induced by 3 nM Arp2/3, 250 µM ATP, 0.1 mM MgCl_2_ and 0.1 mM EGTA, with different amounts of CapZ included in the system (indicated above each image), from clusters made from 500 nM Nck, 250 nM N-WASP and 1.25 nM His_8_-nephrin (15% labeled by Alexa405) on the membrane. Individual images are TIRF image overlays of nephrin-405 shown in cyan and actin-647 shown in magenta. Total Internal Reflection Fluorescence (TIRF) penetration depth 200 nm for all channels. Scale bar, 5 µm. **(B)** Quantification of actin mean and fraction intensity over time for the conditions showed in **(A)**. Actin was selected by selecting the intensity threshold between 250 and 65535 the individual 16-bit images, values for each frame are calculated as described in **Section 4.4**.”

“FIGURE 6 | Representative actin polymerization analysis **(A)** 1 µM actin (5% labeled by Alexa647) polymerization is induced by 3 nM Arp2/3, 6 nM CapZ, 250 µM ATP, 0.1 mM MgCl_2_ and 0.1 mM EGTA, from clusters made from 1 µM Nck, 2 μM N-WASP (15% Alexa488) and 1.25 nM His_8_-nephrin on the membrane. Binary mask from segmentation of the N-WASP images. Pixels outside the clusters are white, pixels inside the clusters are black. Scale bar, 5 µm. **(B)** Quantification of actin polymerization over time for the condition showed in **(A)**. Graph was generated in Matlab. Black dotted lines indicate a linear fit of the five points around the half-max intensity.”

The authors apologize for these errors and state that this does not change the scientific conclusions of the article in any way. The original article has been updated.

